# Fetal and Placental Causes of Elevated Serum Alpha-Fetoprotein Levels in Pregnant Women

**DOI:** 10.3390/jcm13020466

**Published:** 2024-01-14

**Authors:** Joanna Głowska-Ciemny, Konrad Szmyt, Agata Kuszerska, Rafał Rzepka, Constantin von Kaisenberg, Rafał Kocyłowski

**Affiliations:** 1 PreMediCare Prenatal Research Center, 21 Czarna Rola St., 61-625 Poznan, Poland; biuro@premedicare.pl (K.S.); a.kuszerska@cm.uz.zgora.pl (A.K.); biuro@new.med.pl (R.K.); 2 New Med Medical Center, 100 Szamotulska St., 60-566 Poznan, Poland; 3 Department of Gynecology and Obstetrics, Institute of Medical Sciences, University of Zielona Gora, 28 Zyty St., 65-046 Zielona Gora, Poland; r.rzepka@inm.uz.zgora.pl; 4 Department of Obstetrics and Gynecology, Hannover Medical School, Carl-Neuberg-Str. 1, D-30625 Hannover, Germany; vonkaisenberg.constantin@mh-hannover.de

**Keywords:** alpha-fetoprotein, fetal neural tube defects, congenital fetal renal defects, fetal liver disease, fetal gastrointestinal tract defects, fetal genetic skin defects, fetal anemia

## Abstract

The most common association related to alpha-fetoprotein (AFP) is fetal neural tube defect (NTD), and indeed, this is where the international career of this protein began. In times when ultrasonography was not yet technically advanced, the detection of high levels of AFP in maternal serum (MS-AFP) and amniotic fluid was the basis for suspecting neural tube defects. In cases where there was no confirmation of NTD, other causes were sought. It has been established that high titers of MS-AFP could originate in other defects or diseases, such as (1) increased proteinuria in severe fetal kidney diseases; (2) pathological overproduction in liver diseases; (3) penetration through the membranes of gastrointestinal organs exposed to amniotic fluid; (4) passage through the walls of skin vessels; and as a side effect of (5) hepatic hematopoiesis and increased transfer through the edematous placenta in fetal anemia. This article provides a review of the current literature on congenital defects and genetic diseases in the fetus where an elevated level of MS-AFP may serve as the initial diagnostic clue for their detection.

## 1. Introduction

Alpha-fetoprotein (AFP) is a fetal glycoprotein with a mass of 69–70 kDa. Along with genes encoding albumins, alpha-albumins, and vitamin D-3 binding protein, it forms a common gene family located in chromosome 4. AFP has a two-dimensional alpha-helical U-form secondary structure and lacks a beta-helical structure. Spatial conformation comprises three (I–III) homologous domains, each consisting of three spherical subdomains conjugated by 15 regularly distributed disulphide bonds. In the fetus, AFP is initially produced in the yolk sac and subsequently predominantly in the liver, with a small amount also found in the fetal gastrointestinal tract. The maximum concentration of AFP in the fetal serum is reached in the 10th to 13th week of pregnancy, after which it decreases until the time of delivery. In the first year of life, AFP production gradually ceases, and AFP is completely replaced in the newborn’s circulation by albumins. The presence of AFP has also been confirmed in fetal cerebrospinal fluid and amniotic fluid—amniotic fluid alpha-fetoprotein (AF-AFP). In cerebrospinal fluid, AFP primarily appears through filtration by the choroid plexuses of the ventricular system. On the other hand, in amniotic fluid with urine, AFP appears through filtration by the fetal kidneys. In the mother’s serum, AFP appears in the 6th week of pregnancy, after which it decreases until the time of delivery. The presence of AFP in maternal circulation is possible due to transport through fetal membranes in the first trimester of pregnancy and the dominance of transplacental transport (and additionally, to a small extent, through fetal membranes) from the second trimester of pregnancy. We know for certain that the placenta does not produce AFP. Transplacental transport is conditioned by the presence of receptors for AFP in the placenta, which appear from the beginning of the second trimester of pregnancy [1].

A breakthrough discovery in the 1970s was the determination of alpha-fetoprotein levels in the serum of pregnant women to detect fetuses with neural tube defects. In the case of high AFP values in maternal serum, amniocentesis was performed to determine the levels of AFP and acetylcholinesterase (AChE) in the amniotic fluid to confirm the diagnosis with a sensitivity of 75.1% and specificity of 97.7% [2]. Many times, however, despite a positive test result (MS-AFP increased), no NTD defects were found. In those situations, it was only after birth, and with the development of advanced ultrasound techniques and genetic diagnostics, that other disease entities began to be diagnosed on this basis while still in the prenatal period. It has become crucial to understand the origins of alpha-fetoprotein and the process of its circulation between the fetus and mother.

**Figure 1:**
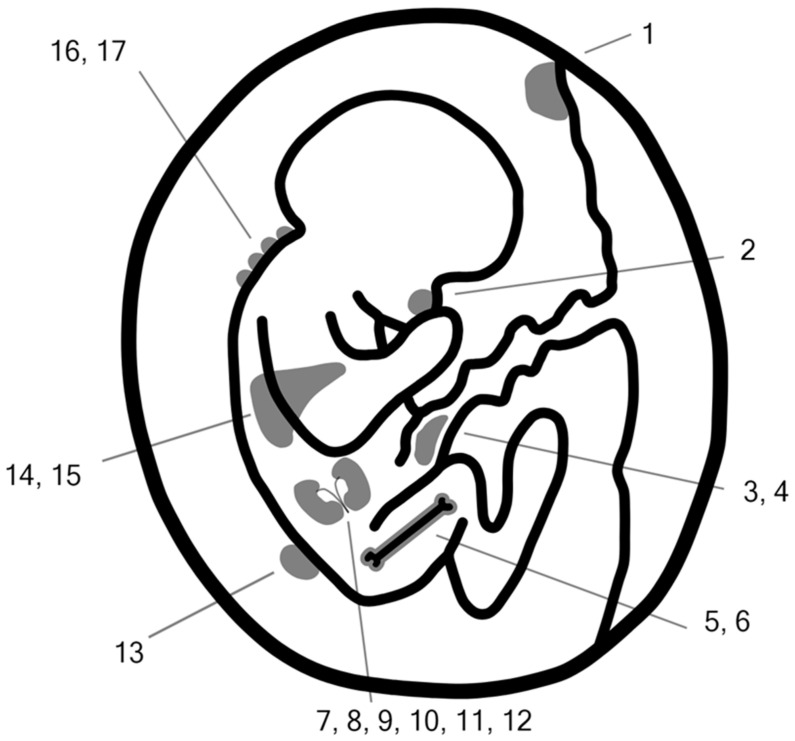
Fetal causes of elevated maternal serum alpha-fetoprotein (MS-AFP) levels during pregnancy: 1. Chorioangioma; 2. Epignathus; 3. Omphalocoele; 4. Gastroschisis; 5. Hemoglobin Barts; 6. Congenital erythropoietic porphyria; 7. Congenital “Finnish type” of nephrotic syndrome; 8. CRB-2-related syndrome; 9. Multiple ACYL-CoA dehydrogenase deficiency; 10. Oculocerebrorenal syndrome of Lowe; 11. Denys–Drash syndrome; 12. Galloway–Mowat syndrome; 13. Neural tube defects; 14. Beckwith–Wiedemann syndrome; 15. Hepatic hemangioma; 16. Aplasia cutis congenita; and 17. Epidermolysis bullosa simplex (see also Table 1).

This article is an overview of fetal diseases, birth defects, and genetic syndromes that can be diagnosed in utero thanks to elevated levels of AFP in maternal serum during the additional use of AFP in prenatal screening, especially in the second and third trimesters (Figure 1) [1,3].

## 2. Methodology

**Figure 2:**
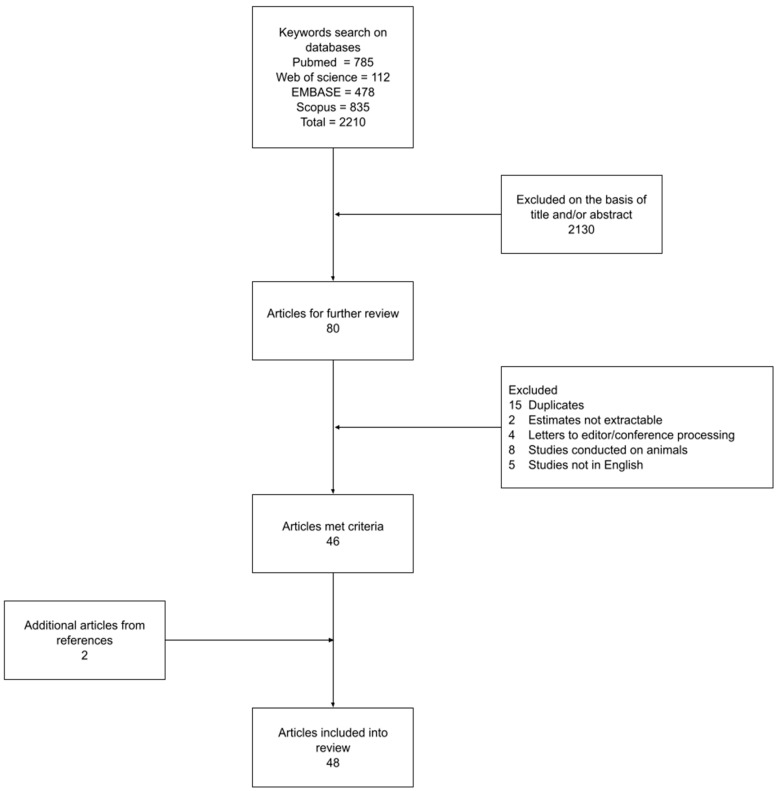
Overview of the search process.

A comprehensive search was conducted on key medical and health databases, including PubMed, Scopus, Web of Science, and Embase, for articles relating to the theme of maternal serum alpha-fetoprotein and congenital defects. Several keywords and their combinations were used to retrieve articles from these databases, including AFP, MS-AFP, fetal anomalies, pregnancy complications, and congenital genetic disorders. Publications that were not in English as well as studies involving animals, duplicates, letters to editors, or conference processing were excluded from the review. Using these search criteria, a total of 48 primary studies were identified that formed the basis of this review (Figure 2).

## 3. Central Nervous System

Intraneuronal synthesis of AFP has never been proven, but its presence in the central nervous system has been confirmed by immunohistochemical studies. It enters the cerebrospinal fluid through the process of blood filtration in the choroid plexuses of the ventricular system. In case of open neural tube defects, AFP leaks with cerebrospinal fluid from the defective structures into the amniotic fluid and is then absorbed through the fetal membranes into the maternal bloodstream. Maternal-serum alpha-fetoprotein (MS-AFP) determination can detect 65–80% of open neural tube defects (88% of anencephaly, where we typically find values around 6.5 multiple of the median (MoM), and 67% of meningo-spinal hernias, where we typically find values around 3.8 MoM). The highest sensitivity in detecting NTDs occurs when AFP testing is performed between 15–18 weeks of gestation, and declines thereafter, as AFP production and concentration also physiologically decline. The usefulness of AFP determination in closed neural tube defects such as cerebral hernia (encephalocele), cleft occipitalis (iniencephaly), or caudal regression syndrome (CRS) has not been demonstrated, as there is no CSF leakage there. Research is currently underway on other non-invasive NTD markers—glial fibrillary acidic protein (GFAP) and complement system proteins. GFAP is a protein involved in the structure of the glial cytoskeleton and a specific marker of CNS defects. Its sensitivity does not decrease with increasing gestational age and is not present in other defects such as evisceration. It also does not increase in cases of closed NTDs. Complement components, in turn, act as conductors of cells to their target sites in the CNS during embryogenesis. Imbalances in this system are thought to be a cause of NTDs (C3 and C9 levels are significantly reduced in NTDs), schizophrenia, or autism [3,4,5,6,7,8,9].

Meningomyelocele (MMC). It occurs with a frequency of 0.4:1000 births. The underlying cause is a genetic (mutations of dozens of different genes)–environmental interaction (folic acid deficiency, among other things). Due to incomplete closure of the neural tube, there is an exposure of the neuroepithelium to amniotic fluid, resulting in neuronal degeneration. In the perinatal period, MMC manifests as a tumor of the spinal region, most often of the L-S segment of the spine, and Arnold–Chiari syndrome (wedging of the cerebellum, fourth ventricle, and brainstem into the great aperture of the skull and consequent hydrocephalus). After birth, hydrocephalus, fecal and urinary incontinence, and flaccid paresis of the lower extremities are the predominant problems [6,7].

**Figure 3:**
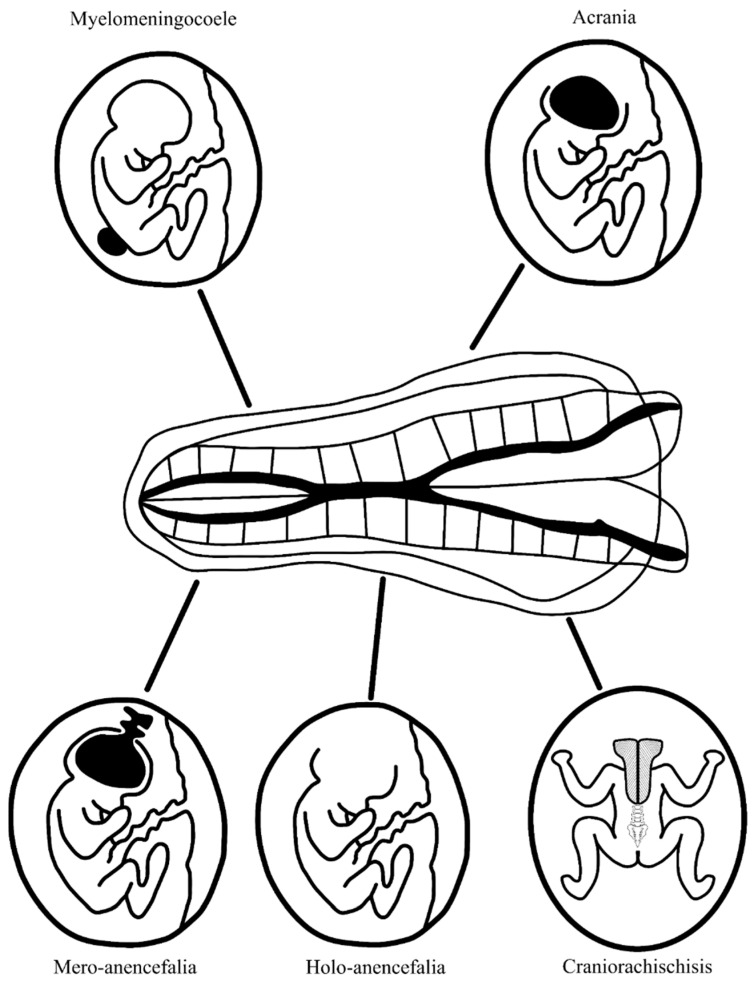
Fetal neural tube defects associated with increased maternal serum alpha-fetoprotein (MS-AFP).

Acrania and anencephaly. They occur with a frequency of 1:10,000–20,000 births. A genetic mutation in the gene encoding hedgehog acyltransferase (HHAT) underlies this condition, which is essential for the production of Hedgehog (Hh) protein, important in the production of kinases that regulate extracellular signals, bone morphogenetic proteins, and fibroblast growth factors [10]. This leads to abnormal bone and cartilage tissue formation. Another cause of acrania can be amniotic band syndrome. This results in the underdevelopment or absence of cranial plates, either partially or entirely. The brain is exposed to amniotic fluid, which eventually leads to the destruction of its structures. There are three forms of anencephaly. Mero-anencephaly involves a small midline gap in the cranial vault with protrusion of immature brain tissue (this is the cerebrovascular area—primitive brain tissue with fields of irregular vascularization) that is not covered by meninges and skin. In holo-anencephaly, the classic form of anencephaly, there is a complete absence of brain tissue except for the brainstem. In craniorachischisis, the most severe form, anencephaly is accompanied by spina bifida, a split in the spinal column to the thoracic level, and the resulting space is filled with the cerebrovascular and medullovascular areas [6,7]. A summary of fetal CNS defects associated with elevated maternal serum AFP levels is shown in Figure 3.

## 4. Liver

From the 26th day after ovulation, the hepatic diverticulum of the archenteron produces AFP; from the 9th week of pregnancy until birth, the fetal liver is the main source of AFP in the fetal bloodstream [1].

Beckwith–Wiedemann Syndrome (BWS). It occurs with a frequency of 1:136,000 births. It is genetically associated with hypermethylation of the H19 gene on chromosome 11p.15.5. During prenatal development, it is characterized by macrosomia, umbilical hernia, macroglossia, organomegaly, polyhydramnios (swallowing disorder), and enlargement of the placenta. Overall, 5–10% of children have a risk of developing embryonal malignancies in childhood, such as Wilms tumor, hepatoblastoma, adrenocortical carcinoma, gonadoblastoma, neuroblastoma, and rhabdomyosarcoma. Elevated AFP levels result from abnormal liver function and leakage through the enlarged placenta. High AFP also persists after birth and is at higher levels than in healthy children. AFP measurement should be used for oncologic monitoring for hepatoblastoma (96% of children with hepatoblastoma have high AFP), based on special nomograms for children with BWS rather than healthy children (characterized by a different, slower rate of descent of AFP levels in the first year of life) [11,12,13,14].

Hepatic hemangioma. It occurs with a frequency of 5:1,000,000 births. It is the most common benign liver tumor in fetuses and newborns. It undergoes a phase of rapid proliferation during infancy and then regression from 1–5 years of age. It can have a focal, multiple, or diffuse form. Most cases are asymptomatic, but heart failure or signs of hepatitis can rarely occur. It has been suggested that the neoplasm itself does not produce AFP, but the interaction between the primary mesoderm, from which it originates, and the endodermal elements from the yolk sac located in the liver is the source [15,16].

## 5. Gastrointestinal Tract

AFP is produced in trace amounts by cells of the gastrointestinal tract from the 26th day after ovulation and comes mainly from the endodermal cells of the middle and posterior archenteron. In the gastrointestinal tract, AFP is also absorbed from ingested amniotic fluid. Postnatally, AFP may serve as a marker of gastrointestinal mucosal regeneration following intestinal surgeries for necrotizing enterocolitis (NEC) or other intestinal injuries in children. These possibilities are associated with endodermal stem cells that persist beyond the fetal period of life [1,17].

Gastroschisis. It occurs with a frequency of 2–5:10,000 births. It is not associated with genetic mutations but rather with a mother’s young age and propensity to use stimulants and drugs. Typically, a small 2–4 cm opening is found to the right of the umbilicus through which the intestines (and sometimes also the liver or stomach) protrude into the amniotic cavity without being covered by any additional membrane. After birth, the main issue is short bowel syndrome [18,19,20]. A particular form of gastroschisis is Limb–Body Wall Complex (body stalk anomaly), where there is both thoracic and abdominal evisceration, limb abnormalities, and encephalocele [18]. The source of high AFP levels in amniotic fluid and maternal serum is the release of AFP from the exposed intestines.

Umbilical hernia (omphalocele) occurs with a frequency of 1:4000 births. It is associated with chromosomal aberrations, mainly trisomy 13 and 18, as well as various genetic syndromes such as Beckwith–Wiedemann syndrome. The hernia sac protrudes from the umbilical ring and has an umbilical cord attachment to its top. It contains the intestines and/or the liver [20]. If the umbilical hernia coexists with pathologies of the abdominal wall below the umbilicus, it is referred to as OEIS complex (omphalocele, bladder exstrophy, imperforate anus, spinal cleft) [21]. When abdominal and chest wall pathologies coexist, this is referred to as pentalogy of Cantrell (umbilical hernia, diaphragmatic hernia, sternal cleft, cardiac ectopy, cardiac defect) [22,23]. Due to the thick membrane coverage, AFP levels in amniotic fluid and serum may be low, and certainly significantly lower than in gastroschisis.

Epignathus. It occurs with a frequency of 1:20,000–40,000 births, with a predominant incidence in women. The genetic etiology is unclear, but it is associated with trisomy 13, ring chromosome X, and pentasomy of the X chromosome. It is a rare teratoma (germinal cell tumor), with most cases occurring in the sacrococcygeal region during fetal life, and only about 2–9% involving the head and neck area. Epignathus can originate in the mandible, maxilla, palate, fetal throat, and sphenoid or ethmoid sinuses. It contains cells of all three germ layers: ectoderm, endoderm, and mesoderm. It can reach massive sizes, protruding in front of the oral cavity and causing airway obstruction after birth (80–100% risk of perinatal death). The tumor is accompanied by deformation of the craniofacial structures (often cleft palate) and a risk of invasion into the brain structures. Impaired swallowing of amniotic fluid leads to polyhydramnios. High AFP levels result from its production by the huge mass of tumor tissue. Monitoring AFP levels in teratomas is used to detect recurrence or progression toward malignancy [24,25].

## 6. Kidneys

AFP production has not been found in the kidneys, neither in the prenatal period nor in childhood. However, congenital abnormalities of nephron structure, particularly the filtration membrane, can cause significant proteinuria, leading to high concentrations of AFP in amniotic fluid and maternal serum [1].

Congenital “Finnish type” of nephrotic syndrome. It occurs with a frequency of 1:8000 births in the Finnish population. It is inherited in an autosomal recessive manner due to mutations in the NPHS1 gene encoding nephrin, a protein on the filtration membrane of kidney glomeruli. In fetal life, there are no apparent anatomical changes, although these children already exhibit significant proteinuria. After birth, severe nephrotic syndrome, malnutrition, and susceptibility to infection are diagnosed. The only treatment is kidney transplantation. High AFP levels in amniotic fluid and maternal serum are typical for both homozygotes and heterozygous carriers. However, carriers of the mutation have elevated AFP levels only up to about the 20th week of gestation. This normalizes in the late second trimester due to the completion of glomerular formation and simultaneous withdrawal of proteinuria [26,27].

Steroid-resistant nephritic syndrome associated with CRB2 mutation. It occurs with a frequency of <1:1,000,000 and is inherited in an autosomal recessive manner. It mimics the congenital “Finnish type” of nephrotic syndrome. Histopathological examination reveals obliteration of foot processes of podocytes. Dilatation of the ventricular system of the brain, renal cysts, heterotopia of the gray matter, VSD, Scimitar syndrome, and the ophthalmic disorders retinitis pigmentosa and congenital Leber blindness are diagnosed perinatally [28,29]. 

Multiple Acyl-CoA Dehydrogenase Deficiency (MADD) = MAD deficiency = Glutaric Acidosis Type 2 = Glutaric Aciduria Type 2. It occurs with a frequency of 1–9:1,000,000 births and is inherited in an autosomal recessive manner. It is caused by mutations in the Electron Transfer Flavoprotein Subunit Alpha—*ETFA* (15q23-q25), Electron Transfer Flavoprotein Subunit Beta—*ETFB* (19q13.3-q13.4), and Electron Transfer Flavoprotein Dehydrogenase—*ETFDH* (4q32-q35) genes, which encode the alpha and beta subunits of ETF-Q oxidoreductase, a component of the mitochondrial respiratory chain. The prenatal period is characterized by fetal growth retardation, oligohydramnios, and polycystic kidneys.

There are two known clinical forms: severe MADD (onset in the neonatal period, with or without congenital malformations) and mild MADD (late onset). The severe form is characterized by prematurity and clinical symptoms appearing within the first day of life, including hypoglycemia, hypotonia, hepatomegaly, metabolic acidosis, and death in the first week of life. Congenital malformations such as polycystic kidneys, facial dysmorphia, genital abnormalities, and rocker-bottom foot may also occur. The late onset form manifests in the first months of life with episodes of vomiting, metabolic acidosis, and hypoglycemia, or only in adolescence in the form of a syndrome resembling Reye’s syndrome with ketoacidosis and lipid storage myopathy. High AFP in maternal serum and amniotic fluid is associated with progressive fetal kidney failure, negative acetylcholinesterase, and an abnormal acylcarnitine profile in amniotic fluid [30,31].

Lowe Syndrome (oculo-cerebro-renal syndrome). It occurs with a frequency of 1:500,000 births and is inherited in an X-linked recessive manner. The mutation is located in the OCRL gene at locus Xq26.1. It manifests with cataracts, glaucoma, hypotonia, intellectual disability, and Fanconi syndrome—necrosis of the proximal tubules resulting in acidosis, glucosuria, aminoaciduria, and loss of citrate, uric acid, and protein. High levels of AFP in maternal serum and amniotic fluid result from fetal kidney damage [32,33].

Denys–Drash Syndrome. Less than 300 cases have been described worldwide. It is inherited in an autosomal dominant manner, mainly due to de novo mutations resulting from loss of genetic material at 11p13 locus, where the suppressor gene Wilms Tumor 1 (WT1) is normally located. It manifests as a triad of nephrotic syndrome, up to end-stage renal failure, Wilms’ tumor, and abnormal genital differentiation. High maternal and amniotic fluid AFP levels result from protein loss due to fetal nephrotic syndrome [34,35].

Galloway–Mowatt Syndrome. It occurs with a frequency of 1:1,000,000 births. It is inherited in an autosomal recessive manner, caused by mutations in the WDR73 gene on chromosome 15 at locus q25.2. This gene is responsible for encoding podocyte-building proteins (GEPP-1, synaptopodin, and nephrin) and the basement membrane (laminins and integrins). Clinical features include microcephaly, esophageal hiatal hernia, nephrotic syndrome, and psychomotor developmental delay. High maternal and amniotic fluid AFP levels result from protein loss due to fetal nephrotic syndrome [36,37].

## 7. Skin

Epidermolysis Bullosa Simplex. It occurs with a frequency of 2:100,000 births. It is caused by mutations in 16 different genes. Most cases of EB simplex are inherited in an autosomal dominant manner, with mutations affecting the KRT5 and KRT14 genes. However, in about 5% of simplex-type EB cases, the disease is inherited in an autosomal recessive manner and caused by mutations in the KRT14 gene. The remaining forms of EB of the simplex type caused by mutations in other genes (ITGA6, ITGB4, DSP1, PKP1) are inherited in an autosomal recessive manner. An exception is the PLEC1 gene, mutations of which can occur in both the autosomal dominant and recessive inherited subtype of EB simplex. The underlying histopathology is an abnormal junction of the epidermis and dermis, resulting in the formation of blisters due to mechanical trauma, which then rupture, leaving erosions and scars. The formation of blisters on the hands and feet, along with their scarring, can lead to contractures. Narrowing of the gastrointestinal tract, urinary tract, and lungs can also occur. High levels of AFP result from its release into the amniotic fluid from exposed blood vessels that would normally be covered by the skin [38,39,40].

Aplasia cutis congenita. It occurs with a frequency of 1:10,000 births. The etiology is heterogeneous and can include occasional mutations in the BMS1 (10q11.21) and DLL4 (15q15.1) genes, as well as the influence of drugs, narcotics, and herpes virus infections. The syndrome is characterized by the absence of skin, typically from the scalp, although other areas may be affected as well. The high AFP levels are due to its release into the amniotic fluid from exposed vessels that would normally be covered by the skin [41,42].

## 8. Hematopoietic System

The main source of AFP is multipotent progenitor cells in the bone marrow. Two subtypes of these cells are described: (1) fetal hepatic stem/progenitor cells (FHSCs) and (2) intrinsic hematopoietic stem/progenitor cells (HSPCs). HSPCs can also migrate to the liver and act as precursors of hepatic stem cells. Another source is bone marrow mesenchymal cells with the ability to differentiate into hepatic stem cells and migrate to the liver in case of damage [1,43].

Hemoglobin Barts (Hb Barts). It occurs with a frequency of 1:200–1:2000 births and is the most common cause of fetal anemia in Southeast Asia. It is the most severe form of alpha-thalassemia. It is inherited in an autosomal recessive manner and results from a deletion in the HBA1 and HBA2 genes (16p13.3). The mutation leads to an abnormal structure of hemoglobin, which consists of four gamma-globin chains (Hb Barts) instead of alpha-globin in fetal life and four beta-globin chains after birth (HbH). Hb Barts and HbH have a high affinity for oxygen but do not release it to the tissues, resulting in anemia and tissue hypoxia, hepatosplenomegaly, placentomegaly, generalized edema appearing before 10 weeks of gestation, and intrauterine or perinatal death. Most children rescued with the help of intrauterine transfusions require lifelong intensive blood transfusions. Elevated maternal serum AFP results from increased fetal–maternal transfer through the edematous placenta and/or from increased extramedullary hematopoiesis in the liver. The increase in MS-AFP levels precedes the increase in MCA-PSV by approximately 2.7 weeks [44,45].

Congenital erythropoietic porphyria. It occurs with a frequency of 1:1,000,000 births and is inherited in an autosomal recessive manner. The most common mutation in the severe form is C73R, resulting in a severe deficiency of uroporphyrinogen III synthase and the accumulation of uroporphyrinogen I and coproporphyrinogen I, followed by accumulation of uroporphyrins and coproporphyrins. In fetal life, it presents as fetal anemia and edema. After birth, it is characterized by bone and teeth discoloration, hemolysis, hepatosplenomegaly, dark urine, photosensitivity resulting in skin wounds, scarring and blisters, alopecia areata (patchy hair loss), keratoconjunctivitis up to blindness, and bone marrow proliferation causing bone fragility and secondary bone fractures. Elevated AFP levels result from fetal liver dysfunction and leakage through the edematous placenta [46,47].

## 9. Placenta

It has been demonstrated that the human cytotrophoblast already produces AFP in the early stages of pregnancy (the transient presence of the AFP gene has been confirmed) before its production begins in the yolk sac at 6 weeks gestation. At the time of delivery, the concentration of AFP in the intervillous space of the placenta is higher than in maternal serum and similar to the concentration in the umbilical cord. During this period of pregnancy, this results from the transfer from the fetal blood through the villi of the placenta. In the case of a normally developed fetus, specific situations such as damage to the syncytiotrophoblast of the placental villi or vessels passing through the basal plate (larger placental volume, abnormal basal plate structure, chronic inflammation of the villi, and placental infractions) can lead to increased leakage of AFP from the fetus to maternal serum [48,49].

Placental hemangioma (chorioangioma) is the most common benign tumor of the placenta. Up to a diameter of 4–5 cm, it has no clinical significance and is asymptomatic. However, large tumors can cause polyhydramnios, hemolytic anemia, thrombocytopenia, and fetal heart failure. The tumor is typically cystic and located peripherally near the placental attachment of the umbilical cord. There are two histopathological types: 1/angiomatous, composed of numerous small blood vessels, and 2/cellular, with poor vascularization. The abnormal thin-walled vessels release AFP into the amniotic fluid and directly into the maternal bloodstream through the placenta [50,51].

## 10. Discussion

The determination of AFP in serum is a relatively inexpensive and widely available test but it lacks specificity. After years of research on AFP, we know that an elevated level in maternal serum may result not only from fetal disorders but also from a neoplastic process in the pregnant woman, such as hepatocellular carcinoma (HCC), germ cell tumors, or hereditary persistence of AFP (HPAFP) [52,53]. There are no limitations for performing AFP during pregnancy, as in the case of, for example, the double marker test. Thanks to numerous studies over the years, we know the trends of concentration in the serum of pregnant women in individual trimesters of pregnancy [54]. However, there is a lack of standardized reference values for specific populations. Moreover, most studies focus on values from the second trimester (obtained as a part of the triple test), less frequently the first trimester, and occasionally the third trimester. Today, it is known that the AFP level in serum is susceptible to the influence of individual factors such as race, BMI, maternal chronic diseases, method of conception, infections, or medications taken during pregnancy, etc. [55,56]. In light of the strong development of diagnostic and genetic techniques, several questions need to be raised about the future of biochemical markers, including AFP.

In the era of increasingly sophisticated, high-resolution ultrasound devices, do we still need biochemical tests to detect congenital anomalies? In the 1970s and 1980s, the sensitivity of ultrasound examinations in detecting neural tube defects was so low that the combination of AFP and acetylcholinesterase determination in amniotic fluid was the primary diagnostic marker for NTD [57,58]. Currently, we have high-quality ultrasound equipment and well-trained ultra-sonographers, allowing us to diagnose neural tube defects (NTDs) with a high sensitivity of 97% and specificity of 100% using only ultrasound [59]. This has eliminated the need for entirely unnecessary invasive tests, such as confirming high AFP levels in amniotic fluid, as the definitive diagnosis for NTD. In 1999, Ventizileos et al. conducted a cost-effectiveness analysis comparing the determination of MS-AFP and ultrasound examination in detecting neural tube defects (NTDs) in the second trimester of pregnancy. They found that ultrasound is more cost-effective than AFP when the effectiveness of NTD detection in ultrasound exceeds 51% [60]. As is known, not every obstetrician worldwide has access to high-quality ultrasound equipment or the skills to operate it. Therefore, additionally measuring the AFP level in serum can at least raise suspicion, for example, of a neural tube defect, and guide the pregnant woman to a reference center, helping avoid legal consequences of not detecting the defect.

Is there a place for biochemical markers in current conditions, where every patient with suspected genetic syndrome after an ultrasound examination can be offered at least non-invasive prenatal testing (NIPT)? NIPT detects a limited range of genetic abnormalities (trisomies, sex chromosome aberrations, and single microdeletions). It should not be proposed independently without ultrasound examinations, which rule out anomalies, growth disturbances, abnormalities typical of twin pregnancies, or placental pathologies [61]. NIPT is an expensive test, inaccessible to many patients, and reimbursed only in some countries, such as Belgium, the Netherlands, or Germany. What is prospectively beneficial are the continuously decreasing cost, the expansion of diagnostic capabilities, and the possibility of performing it throughout the entire pregnancy [62]. NIPT is also better accepted by patients than biochemical tests, but it is not without drawbacks. It has a limited diagnostic scope and often provides ambiguous results, and sometimes even false positives. NIPT can be falsely positive in situations such as placental mosaicism [63,64], maternal neoplastic diseases (cases of rare autosomal trisomies), or a dead fetus—vanishing twin [62]. In summary, NIPT is not an ideal test; it is also only a screening test with a high cost, lacks reimbursement in many even affluent countries, has a limited diagnostic scope, and requires the verification of a positive result through invasive testing.

**Figure 4:**
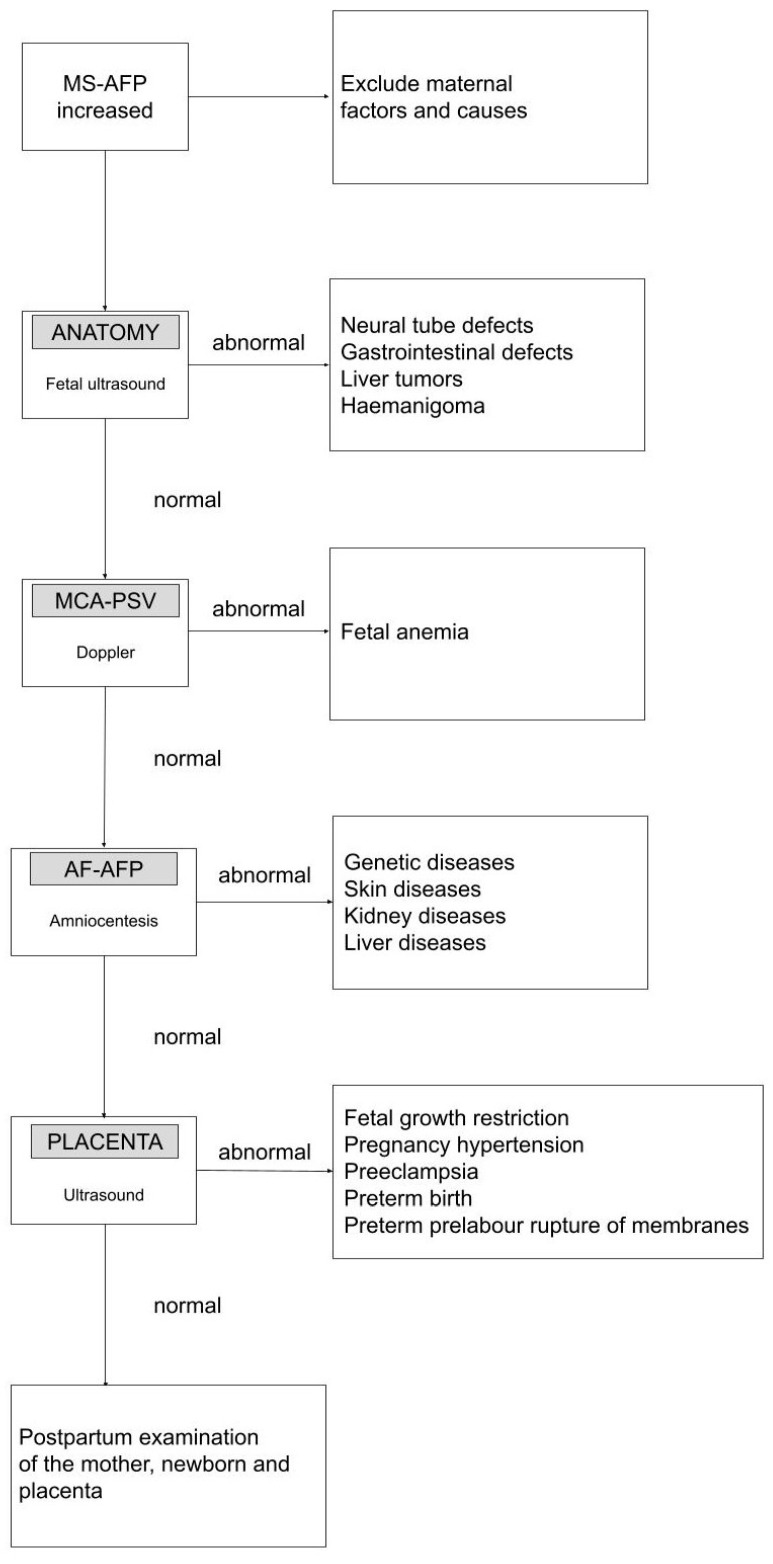
Clinical management protocol of elevated maternal serum alpha-fetoprotein (MS-AFP).

Thus, is there a place for AFP in the medicine of the future? Currently, the main trend in AFP research is an attempt to use it in predicting adverse obstetric outcomes. In first-trimester screening, the proteins PAPPA-A and PIGF have been successfully utilized to predict the risk of preeclampsia and apply acetylsalicylic acid prophylaxis. It is the only adverse obstetric event for which there is preventative care. Why not explore further possibilities for preventive actions? In cases of elevated MS-AFP levels without evident fetal abnormalities in ultrasound or genetic anomalies, suspicion arises about a poorly implanted or malfunctioning placenta (with damaged vascular structures and excessive permeability to AFP), leading to increased absorption into the bloodstream of the pregnant woman [65]. Currently, AFP is tested both independently and in combination with other biochemical markers (PAPPA-A, beta-HCG, estriol, inhibin-A, PIGF, and sFIt-1) in early diagnoses of preeclampsia, small for gestational age (SGA), preterm birth (PTB), and premature prelabor rapture of membranes (PPROM), as well as placenta accreta spectrum (PAS) (Figure 4). While there is no doubt about the utility of AFP results from second-trimester studies in this regard, the usefulness of first-trimester results raises doubts. According to studies by Hu et al. and Melamed et al., elevated AFP values in the first trimester of pregnancy are not useful as standalone diagnostic markers for adverse pregnancy outcomes (APOs) and should not lead to excessive obstetric surveillance [66,67].

Considering the dilemmas above, AFP will be a valuable addition to the diagnosis of congenital anomalies in countries with limited access to ultrasound and genetic tests. In highly developed countries, like other biochemical markers in obstetrics, AFP is slowly fading into obscurity in the era of high-sensitivity testing. It seems that the future of AFP will primarily involve assessing its utility as one of the markers for adverse obstetric outcomes.

## 11. Conclusions

1. AFP is useful in the diagnosis of fetal open neural tube defects, where there is leakage of cerebrospinal fluid into the amniotic fluid (as opposed to closed defects) and it is then absorbed into the bloodstream of the pregnant woman, causing elevated MS-AFP.
2. In fetal liver diseases, elevated MS-AFP levels result from abnormal liver function and leakage through the enlarged placenta.
3. In fetal gastrointestinal defects, AFP enters the amniotic fluid through the wall of organs, mainly the intestines, and then gets absorbed into the bloodstream of the pregnant woman.
4. Fetal kidney diseases with damage to the filtration membrane of nephrons cause increased loss of serum proteins (including AFP) into the amniotic fluid and then into the bloodstream of the pregnant woman.
5. AFP is released into the amniotic fluid along with transudates from pathologically exposed fetal skin vessels and fetal tumor or placental tissues.
6. In fetal anemia, elevated AFP levels are due to the activation of hepatocytes in hepatic hematopoiesis, leading to its increased production and leakage through the swollen placenta into the maternal circulation.
7. In placental tumors, the elevated AFP levels in maternal serum are caused by its release into both the amniotic fluid and the maternal placental circulation.

## Figures and Tables

**Table 1:** Mechanisms of pathological increase in AFP levels in pregnant women in various disease entities (increased fetal production and/or fetal–maternal leakage, + means Yes, − means No); see also Figure 1.

	Disease Entity	Increased Production	Increased Leakage
1.	Chorioangioma	−	+
2.	Epignathus	+	−
3.	Omphalocele	−	+
4.	Gastroschisis	−	+
5.	Hemoglobin Barts	+	+
6.	Congenital erythropoietic porphyria	+	+
7.	Congenital “Finnish type” of nephrotic syndrome	−	+
8.	Steroid-resistant nephritic syndrome associated with CRB2 mutation	−	+
9.	Multiple acyl-CoA dehydrogenase deficiency (MADD)	−	+
10.	Lowe syndrome	−	+
11.	Denys–Drash syndrome	−	+
12.	Galloway–Mowatt syndrome	−	+
13.	Myelomeningocele	−	+
13.	Acrania	−	+
13.	Anencephaly	−	+
14.	Beckwith–Wiedemann syndrome	+	+
15.	Hepatic hemangioma	+	−
16.	Aplasia cutis congenita	−	+
17.	Epidermolysis bullosa simplex	−	+

## Abbreviations

AFP	alpha-fetoprotein
MS-AFP	maternal serum alpha-fetoprotein
AF-AFP	amniotic fluid alpha-fetoprotein
MoM	multiple of the median
NTDs	neural tube defects
AchE	acetylcholinesterase
CRS	caudal regression syndrome
GFAP	glial fibrillary acidic protein
MMC	meningomyelocele
CNS	central nervous system
BWS	Beckwith–Wiedemann syndrome
NEC	necrotizing enterocolitis
OEIS	omphalocele–exstrophy–imperforate anus–spinal defects
MADD	multiple acyl-CoA dehydrogenase deficiency
EB	epidermolysis bullosa
FHSC	fetal hepatic stem/progenitor cells
HSPC	hematopoietic stem/progenitor cells
MCA-PSV	peak systolic velocity in the middle cerebral artery
HCC	hepatocellular carcinoma
HPAFP	hereditary persistence of alpha-fetoprotein
